# Canine B Cell Lymphoma- and Leukemia-Derived Extracellular Vesicles Moderate Differentiation and Cytokine Production of T and B Cells In Vitro

**DOI:** 10.3390/ijms23179831

**Published:** 2022-08-29

**Authors:** Magdalena Zmigrodzka, Olga Witkowska-Pilaszewicz, Rafał Pingwara, Aleksandra Pawlak, Anna Winnicka

**Affiliations:** 1 Department of Pathology and Veterinary Diagnostics, Institute of Veterinary Medicine, Warsaw University of Life Sciences (WULS-SGGW), Nowoursynowska 159c, 02-787 Warsaw, Poland; 2 Department of Large Animal Diseases and Clinic, Institute of Veterinary Medicine, Warsaw University of Life Sciences (WULS-SGGW), Nowoursynowska 159c, 02-787 Warsaw, Poland; 3 Department of Physiological Sciences, Institute of Veterinary Medicine, Warsaw University of Life Sciences (WULS-SGGW), Nowoursynowska 159c, 02-787 Warsaw, Poland; 4 Department of Pharmacology and Toxicology, Faculty of Veterinary Medicine, Wroclaw University of Environmental and Life Sciences, CK Norwida 31, 50-375 Wroclaw, Poland

**Keywords:** extracellular vesicles, CLB70, oncosomes, lymphocytes, dog, leukemia, lymphoma, proliferation

## Abstract

Extracellular vesicles (EVs) are formed in physiological and pathological conditions by almost all mammalian cells. They are known as submicron “molecules” that transport and horizontally transfer their cargo from maternal cells to donor cells. Moreover, cancer cells produce tumor-derived EVs (TEVs), which are present in blood of patients with solid tumors and those with hematological malignancies. Their role in evading immune system surveillance and induction of immunosuppression in hematological cancer is limited. According to the authors’ best knowledge, there is no information about the impact of TEVs from canine lymphoma (CLBL-1) and leukemia (CLB70) on lymphocytes isolated from peripheral blood mononuclear cells (PBMCs). In conclusion, we demonstrate in in vitro experiments that CLBL-1 EVs and CLB70 EVs are effectively taken up by T and B lymphocytes. TEVs decrease the percentage of B lymphocytes and increase that of T lymphocytes, and change T cells’ phenotype into the effector memory (EM) or terminally differentiated effector memory (TEMRA) subtype after in vitro co-culturing. Moreover, CLBL70 EVs have pro-tumorogenic properties by inhibiting the production of CD8^+^IL-17^+^ cells.

## 1. Introduction

One of the evolutionary oldest mechanisms of communication by almost all cell types is the release of extracellular vesicles (EVs). These interact with target cells at close or distant sites and modify their functions [1,2,3]. EVs are classified based on their size and their maternity [4,5,6]. In recent years, terminology of EVs based on their size has been used and the following categories have been distinguished: exosomes (EXSMs) (30–150 nm), ectosomes (ECTSMs) (150–500 nm), and apoptotic bodies (ApoBDs) (800–5000 nm) [7,8]. However, since 2018, as a result of a consensus of the International Society of Extracellular Vesicles (ISEV), the classification of EVs as small, with a diameter below 200 nm, and medium or large, with a diameter over 200 nm, has been recommended [2]. EVs are isolated from blood and body fluids such as urine, semen, breast milk, or cerebrospinal fluid [9,10,11]. EVs have a unique content of cargoes from their donor cells [2,3,12]. They contain moieties of surface proteins, glycoproteins, maternal surface receptors (CD61-platelets, CD14-monocytes, CD3-Tlymphocytes), enzymes (enolases, phosphoglycerate kinase, aldolases), phosphatidylserine, tetraspanins (CD9, CD63, CD81), and MHCI and MHCII molecules. Moreover, they carry translation initiation factor, eIF4E, and elongation factor, eEF1. The mRNA carried by EVs is functional, which means it is capable of encoding polypeptides in support of protein synthesis. Interestingly, some miRNAs that have been found in EXSMs were expressed at higher levels than in the donor cells [9,13,14].

Cancer cells (CCs) express proteins or their mutant forms and other specific biomolecules, and release EVs with their unique “cancer” cargo. Regularly they are named as oncosomes (Os) or large oncosomes (LOs) with sizes of 100–400 nm and 1–10 μm, respectively [15,16]. Os and LOs, like other EVs, are released throughout circulation, but their effect on proximal and distal tissue in hematological malignancies is still not adequately described [17]. Companion animals such as dogs and cats share a common environment with humans, and spontaneously occurring hematological malignancies in dogs have strong similarities with human ones. Moreover, like in humans, lymphoma and leukemia are regarded as being among the most important chronic diseases, and shorten the longevity and quality of patients’ life [18]. Ranked behind mammary gland and skin tumors, lymphoma represents the third most common spontaneously occurring type of tumor diagnosed in dogs [18,19]. More than 80% of all lymphoid malignancies are lymphoma [20,21]. Additionally, chronic lymphocytic leukemia (CLL), acute lymphoblastic leukemia (ALL), and stage V lymphoma have lymphocytosis as a primary feature; hence, the distinction among these diseases can be sometimes unclear [22]. Establishment of two new canine cell lines—CLBL-1 (from the most common canine lymphoma, i.e., the diffuse B cell lymphoma) and CLB70 (chronic lymphocytic leukemia from mature B cells)—and in vitro experiments within these cell lines, may promote translational and comparative lymphoma/leukemia research in humans and dogs [23]. In previous work, we showed that, similarly to humans, platelet derived extracellular vesicles (PEVs) comprised the most numerous population of EVs in healthy dogs and in dogs with cancer [24]. Moreover, the number of T cell EVs (CD3^+^) was higher in dogs with neoplasm and the highest number was observed in dogs with T cell lymphoma and dogs with diffuse B cell lymphoma. The highest number of leukocyte- derived EVs (CD45^+^) was counted in dogs with testis adenocarcinoma, and in a bitch with uterus sarcoma with metastasis [24]. TEVs act by horizontal transfer of their cargo, modulating the tumor microenvironment (TME), including lymphocytes and monocytes. To the best of our knowledge, there is no information regarding TEVs’ influence in dogs; thus, the aim of the study was to evaluate the impact of TEVs derived from leukemia and lymphoma cells lines on peripheral blood mononuclear cells (PBMCs).

The first evidence gathered in 1978 showed that, in Hodgkin’s disease, tumor cells shed EVs [25]. Various studies have shown that tumor-derived EVs (TEVs), particularly from solid tumors, are implicated in modulating the tumor microenvironment (TME) and contributing to the inhibition of anti-tumor activity. In myeloid neoplasm, in an autocrine manner, TEVs promote growth and drug resistance of leukemic cells, and modify the bone marrow niche [26,27]. A high level of EVs is observed in patients’ blood samples compared to normal controls in a variety of pathological conditions and hematologic malignancies [28]. Therefore, plasma samples as a source of EVs from patients with various diseases are used as a noninvasive source of liquid biopsies and specific EV cargos for potential diseases markers [29,30].

## 2. Results

### 2.1. CLBL-1 EVs and CLB70 EVs Are Taken up by Lymphocytes

**Figure 1:**
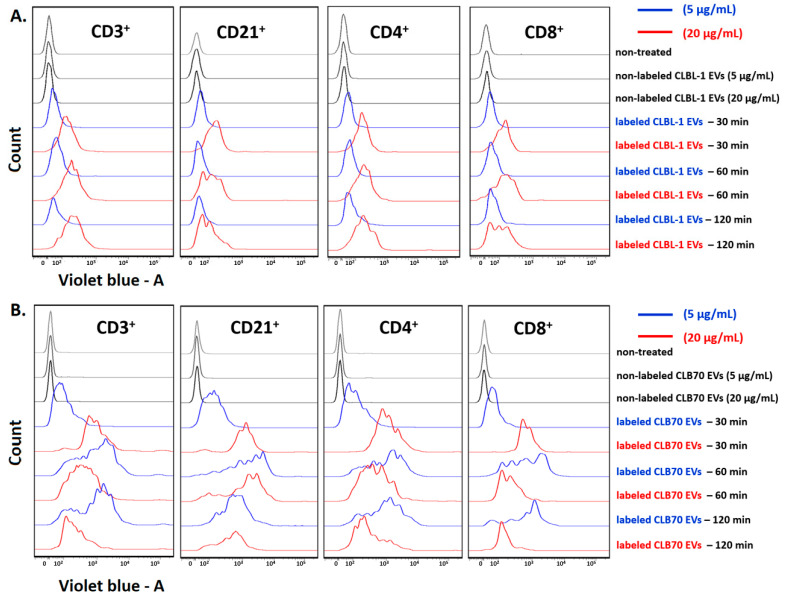
Time-dependent internalization of TEVs by lymphocytes. Flow cytometric analysis of the fluorescence transferred by CLBL-1 EVs and CLB70 EVs into lymphocytes. The staining of directly labeled cells and the negative control cells (gray lines) after 30, 50, and 120 min of incubation are shown. (**A**) the kinetics of two concentrations of CLBL-1 EVs taken up by: CD3^+^ T lymphocytes, CD21^+^ B lymphocytes, CD4^+^ T lymphocytes, and CD8^+^ T lymphocytes. (**B**) The kinetics of two concentrations of CLB70 EVs taken up by: CD3^+^ T lymphocytes, CD21^+^ B lymphocytes, CD4^+^ T lymphocytes, and CD8^+^ T lymphocytes.

To demonstrate the uptake of TEVs (CLBL-1 EVs and CLB70 EVs) by target cells, 5 × 10^5^ PBMCs were exposed for 0.5, 1, and 2 h to CellTrace Violet-labeled TEVs at two concentrations: CLBL-1 EVs and CLB 70 EVs (5 μg/mL) and CLBL-1 EVs and CLB 70 EVs (20 μg/mL) in 0.5 mL medium. Cells were incubated at 37 °C with 5% CO_2_ in the presence of ConA (5 μg/mL). Next, the cells were washed in dPBS, stained with mAbs, and immediately analyzed by flow cytometry. The results confirmed that TEVs are taken up by lymphocytes (Figure 1). Both cell lines’ EV uptake was observed within 30 min of cells’ co-incubation. The effectiveness of CLBL-1 EVs’ internalization was in a concentration-dependent manner, whereas CLB70 EVs were comparable in both 5 and 20 μg/mL concentrations for all subpopulations of lymphocytes. CLB70 EVs were taken up more actively than CLBL-1 EVs.

### 2.2. CLBL-1 EVs and CLB70 EVs Influence on Lymphocyte Subsets and Cytokine Production

To consider the potential impact of taken-up TEVs on T cell function, we co-incubated PBMCs activated by ConA and IL-2, with the presence of CLBL-1 EVs and CLB70 EVs in two concentrations. After 5 days, the intracellular release of specific T cell cytokines IFN-γ and IL-17, and lymphocytes’ proliferative capacity, were analyzed.

#### 2.2.1. CLBL-1 EVs and CLB70 EVs Do Not Influence Lymphocyte Proliferative Capacity

There were no differences in the proliferation of PBMCs in the presence of CLBL-1 EVs and CLB70 EVs compared to PBMCs stimulated only by ConA and IL-2 (*p* > 0.05).

**Figure 2:**
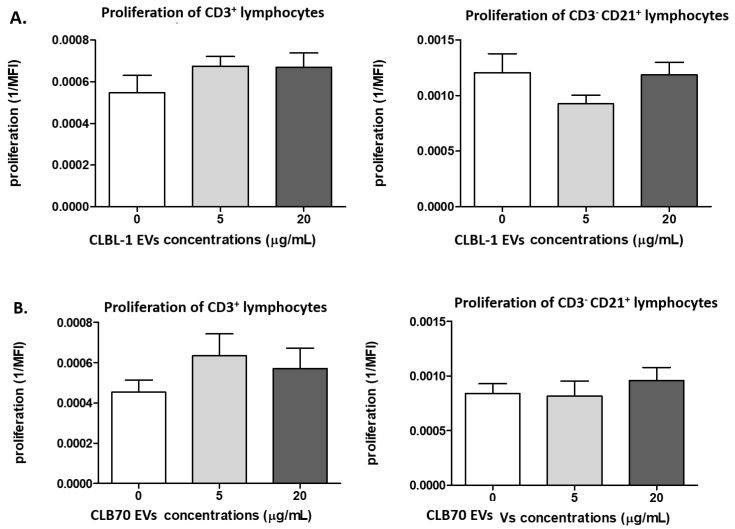
Influence of TEVs on the proliferation of activated lymphocytes. The figures show the proliferation intensity of CD3^+^ and CD3^−^CD21^+^ lymphocytes after 5 days of culture of PBMC in a 37 °C, 5% CO_2_ environment with ConA and IL-2, in the presence of CLBL-1 EVs (**A**) and CLB70 EVs (**B**) in two concentrations or without TEVs (n = 7). The results are presented as the mean ± SEM.

Proliferation of Tlymphocytes (CD3^+^) in the presence of CLBL-1 EVs was found in the control samples (0.0005), CLBL-1 EVs (5 μg/mL) (0.0007), and CLBL-1 EVs (20 μg/mL) (0.0007). For B cells (CD3^−^CD21^+^), proliferation was 0.001, 0.0009, and 0.001 respectively (Figure 2A).

Proliferation of Tlymphocytes (CD3^+^) in samples co-cultured with CLB70 EVs was found in the control samples (0.0004), CLB70 EVs (5 μg/mL) (0.0006), and CLB70 EVs (20 μg/mL) (0.0005). For B cells (CD3^−^CD21^+^), proliferation was 0.0008, 0.0008, and 0.0009 (Figure 2B), and the representative proliferation graph of CD3^+^ and CD3^−^CD21^+^ is shown in Supplementary Figure S1.

#### 2.2.2. CLBL-1 EVs and CLB70 EVs Have an Impact on Lymphocyte Immunophenotype

**Figure 3:**
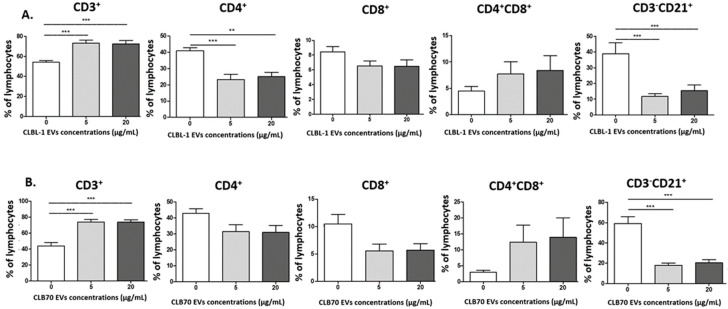
Influence of TEVs on the percentage of subpopulations of activated lymphocytes. The percentage of CD3^+^, CD4^+^, CD8^+^, CD4^+^CD8^+^, and CD3^−^CD21^+^ lymphocytes after 5 days of culture of PBMC in a 37 °C, 5% CO_2_ environment with ConA and IL-2 in the presence of CLBL-1 EVs (**A**) and CLB70 EVs (**B**) in two concentrations or without TEVs (n = 7). The results are presented as the mean ± SEM. The significance levels are: ** *p* < 0.01 and *** *p* < 0.001.

After culturing of PBMCs with CLBL-1 EVs and CLB70 EVs in two concentrations, the percentage of T cells (CD3^+^) was higher in comparison to that of control cells. The Th (CD4^+^) lymphocyte percentage was lower only during co-culturing with CLBL-1 EVs. The B cell (CD3^−^CD21^+^) percentage was lower, regardless of CLBL-1 EV and CLB70 EV concentration, compared to control samples. We observed decreasing tendency in percentage of CD8^+^ cells and increasing tendency in CD4^+^CD8^+^ cells dependent on TEV concentration. Changes in the percentage of T cells and B cells were not dependent on TEV concentration (Figure 3). Next, the CD4^+^/CD8^+^ cells ratio was calculated (Supplementary Figure S2).

**Figure 4:**
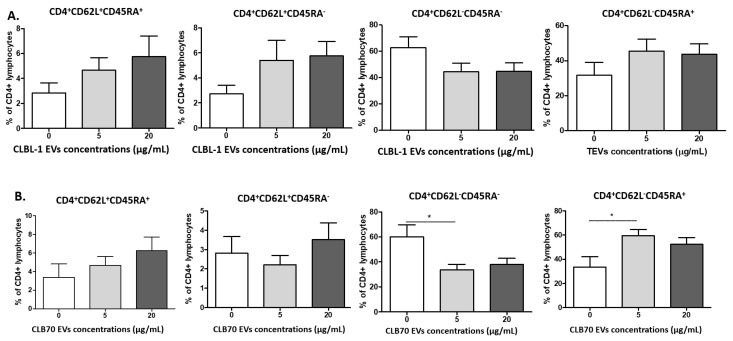
Influence of TEVs on the percentage of activated memory CD4^+^ lymphocytes. Changes in frequencies of canine CD4^+^ lymphocytes after 5 days culture of PBMCs in a 37 °C, 5% CO_2_ environment with ConA and IL-2 in the presence of CLBL-1 EVs (**A**) and CLB70 EVs (**B**) in two concentrations or without TEVs (n = 7). The results are presented as the mean ± SEM. The significance level is: * *p* < 0.05.

The next step was the evaluation of the influence of CLBL-1 EVs and CLB70 EVs on the percentages of CD4^+^ and CD8^+^ naïve, central memory (CM), effector memory (EM), and terminally differentiated effector memory (TEMRA) cells (Figure 4). Their phenotypes were as follows: naïve (CD62L^+^CD45RA^+^), CM (CD62L^+^CD45RA^−^), EM (CD62L^−^CD45RA^−^), and TEMRA (CD62L^−^CD45RA^+^). The percentage of CD4^+^ effector memory cells decreased in the presence of 5 µg/mL of CLB70 EVs, whereas the percentage of CD4^+^ terminally differentiated effector memory cells also increased at that CLB70 EV concentration.

**Figure 5:**
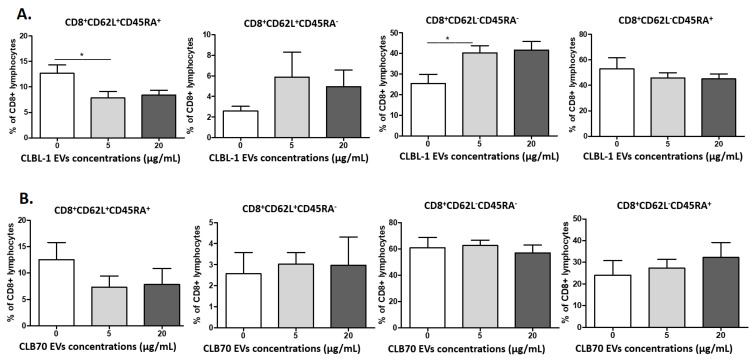
Influence of TEVs on the percentage of activated memory CD8^+^ lymphocytes. Changes in frequencies of canine CD4^+^ and CD8^+^ lymphocytes after 5 days culture of PBMCs in a 37 °C, 5% CO_2_ environment with ConA and IL-2 in the presence of CLBL-1 EVs (**A**) and CLB70 EVs (**B**) in two concentrations or without TEVs (n = 7). The results are presented as the mean ± SEM. The significance level is: * *p* < 0.05.

The percentages of CD8^+^ naïve cells decreased and increased for effector memory cells only in samples co-cultured with CLBL-1 EVs at the concentration of 5 µg/mL (Figure 5).

#### 2.2.3. CLB70 EVs Influence Lymphocytes’ T Cytokine Production

**Figure 6:**
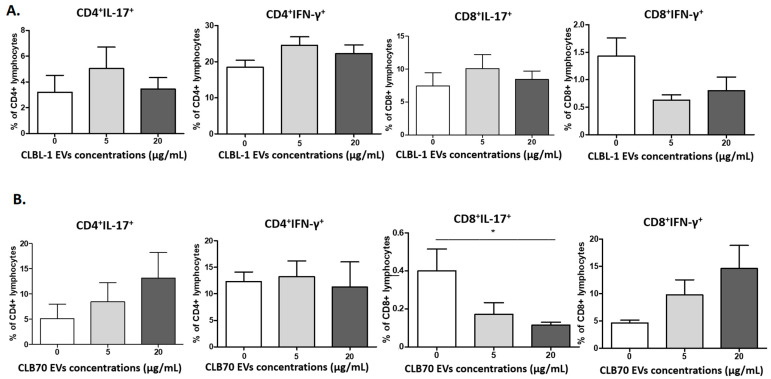
Influence of TEVs on the percentage of IL-17^+^ and IFN-γ^+^ CD4^+^ and CD8^+^ activated lymphocytes. Changes in frequencies of canine CD4^+^IFN-γ^+^ and CD8^+^IFN-γ^+^ and CD4^+^IL-17^+^ lymphocytes after 5 days of culture of PBMCs in a 37 °C, 5% CO_2_ environment with ConA and IL-2 in the presence of CLBL-1 EVs (**A**) and CLB70 EVs (**B**) in two concentrations or without TEVs (n = 7). The results are presented as the mean ± SEM. The significance level is: * *p* < 0.05.

The intracellular production of IL-17 and IFN-γ by CD4^+^ and CD8^+^ lymphocytes was evaluated (Figure 6). There was no influence of CLBL-1 EVs and CLB70 EVs on IFN-γ production (*p* > 0.05); only the intracellular expression of IL-17 decreased for CD8^+^ cells after co-culturing with CLB70 EVs at the concentration of 20 µg/mL.

## 3. Discussion

EVs are known to be evolutionarily conserved mechanisms of intercellular crosstalk. Mammalian cells in physiological and pathological conditions, and CCs, secrete EVs into the intracellular space. Like other soluble factors, such as cytokines or interleukins, these messenger “molecules” modulate immune response [31,32]. The role of TEVs in both immune cell activation and immunosuppression in TME is accepted, but the underlying mechanisms, particularly in malignant diseases, are still unclear. The TME is defined as a heterogenous population of infiltrating and proliferating CCs, resident host cells, fibroblasts, a variety of secreted factors, and extracellular matrix, in addition to blood vessels. Considerable studies have discussed the effect of TEVs derived in solid tumors on the TME but the number of publications that explain the role of lymphoma/leukemia TEVs on the TME and immune cells, and their functions, is limited [33]. Notably, TEVs derived from human tumors inhibit the functions of immune cells, but there is no information about the influence of canine TEVs on their lymphocytes and monocytes.

Presuming TEVs are critical in shaping an inflammatory TME and thereby facilitate disease dissemination to distant organs, we assessed the uptake and functional responses by lymphocyte fraction from PBMC cultures with TEVs isolated from two canine cell lines: CLBL-1 and CLB70. Both types of TEVs, in two concentrations (5 and 20 µg/mL), were effectively internalized by T and B lymphocytes after 30 min of co-culturing. There were no essential differences with the take up of CLBL-1 EVs depending on their concentration, whereas CLBL70 EV internalization was higher in the 20 µg/mL concentration. Moreover, CLB70 EV internalization by T and B lymphocytes, and Th and Tc cells, was higher after 60 min of co-incubation and similar after 120 min, which was not observed in CLBL-1 EVs. In humans, Bennit et al. showed that exosomes from WSU-DLCL2 (DLBCL) are favorably taken up by B cells and monocytes compared to T cells and NK cells, and these findings are in line with those of Hazan-Halevy et al. [34,35]. The most prevalent mechanism of EV internalization is endocytosis, which releases the EV cargo into the recipient cell cytosol [36,37]. However, EVs may change recipient cell functionality without endocytosis via transfer receptors or molecule-activated T cell receptors [38,39]. Notable differences in internalization of CLBL-1 EVs and CL70 EVs may arise from the kinetic accumulation of differences or by the mechanism of transfer of TEV cargo.

There are three main strategies by which TEVs increase the immunosuppressive ability of the TME and favor tumor growth. It is realized by changes in proliferation rate and differentiation of T and B lymphocytes and NK cells [40], and by reprogramming macrophages toward an M2 phenotype, which was described in colorectal cancer, breast cancer, and melanoma [41,42]. Wieckowski et al. showed that TEVs, but not Th cells, inhibit signaling and proliferation of activated CD8^+^ and induced their apoptosis [42]. The upregulated proliferation of Treg cells via TGF-β1 transferred by TEVs was observed in patients with ovarian cancer [43]. Elevated TGF-β1 concentrations in TEVs isolated from AML patients reflected chemotherapy treatment [44]. Shroeder and colleagues showed that EXSMs from head and neck squamous cell carcinoma (HNSCC) inhibit B cell proliferation and increase their apoptosis [45]. Patel et al. showed that ALL-derived TEVs promote proliferation and survival of pre-B acute lymphoblastic leukemia cells [46]. Haque demonstrated that ALL-derived EXSMs regulate in vitro B cell proliferation through the secretion of anti-apoptotic factors [47]. In our study, we did not observe changes in T and B cell proliferation. TEV concentration chosen in our study had to reflect concentrations similar to those in blood of patients with cancer. In the above-mentioned studies, TEV and EXSM concentrations were more than 20-fold higher; thus, it may be the main reason why the proliferation was unchanged in our study.

CD4^+^ T cell subsets are essential in adaptive immunity, and help CD8^+^ T and B cells, which are recruiters of immune cells to the inflammation site and initiate immunological memory [48]. We demonstrated that the percentage of all T lymphocytes increased after 5 days of co-incubation with CLBL-1 EVs and CLB70 EVs in both concentrations compared to the control, but the Th cell percentage was decreased in samples with CLBL-1 EVs in comparison to those of CLB70. The differences in maturity of CCs from CLBL-1 and CLB70 may reflect changes in CD4^+^ percentage. Compared with CLBL-1, the CLB70 cell line is represented by more mature B cells [18]. Shao and colleagues confirmed that EXSMs from multiple myeloma cell lines in humans inhibit CD4^+^ proliferation and promote proliferation and TGF-β secretion by Tregs [49]. The higher percentage of T lymphocytes may be caused by an increased number of Tregs, which was not elucidated in our work. In solid tumors, TEVs induce expansion of Tregs [50]. Similar observations were made in leukemia, increasing tumor growth and modifying TME in an autocrine manner, and modulating the vascular and stromal bone marrow niche in humans [33]. In non-Hodgkin lymphoma, infiltrating Tregs suppress cytotoxic T cells in TME [51].

Changes in lymphocytes’ T function and phenotype are age-related and result in the immunosenescence of older dogs and humans [52]. Thus, in this study, we decided to collect PBMCs only from healthy and young dogs to avoid the impact of age-related diseases on the results. Withers et al. showed decreased frequencies of naive CD4^+^ and CD8^+^ T lymphocytes, and an increased percentage of TEMRA CD8^+^ T lymphocytes in dogs over six years old. These observations revealed that aged dogs displayed features of immunosenescence similar to those of humans and mice [53]. In our study, CLBL70 EVs decreased the percentage of CD4^+^ EM (CD62L−CD45RA−), and increased CD4^+^TEMRA cells (CD62L−CD45RA^+^), whereas CD8^+^ naïve cells decreased and the CD8^+^ EM percentage increased in the presence of CLBL EVs. These results showed that TEVs, depending on their origin, shifted CD4^+^ and CD8^+^ phenotypes and their functions [42]. CLBL 70 EVs decreased the percentage of CD8^+^T lymphocytes with intracellular IL-17 expression. Although IL-17-producing T cells have been shown to be anti-tumorigenic in adoptive T cell therapy settings [48,54], our results confirm the pro-tumorigenic functions of CD8^+^ lymphocytes.

Study limitations: according to good ethical practice the study was not performed in vivo, and the main limitation of the study is connected with the relatively low number of animals studied. In vitro data are therefore essential in gaining basic information that can be translated in vivo. We decided to use young dogs to avoid the possibility of systemic diseases. In addition, all actions were performed to obtain results that were as accurate as possible during all experiments.

## 4. Materials and Methods

### 4.1. Animals and Blood Samples

Eight adult dogs that presented for periodic health examination at a veterinary clinic in Warsaw were included in the study. Only dogs without clinical signs of disease during anamnesis and clinical examination, and without vaccination or treatment two weeks before blood sampling, were accepted for the study. The animal research group consisted of four females (three neutered) and four males, comprising four mixed breeds and one German Shepperd, Greyhound, Border Terrier, and Miniature Schnauzer. The median age of the dogs was 2.3 years (range 1–4.5).

Only excess peripheral blood (about 2 mL) collected for routine diagnostic tests was used for this study. The blood collection was a part of a non-experimental clinical veterinary examination consented to by the owners of dogs; therefore, according to the European directive EU/2010/63 and local regulations regarding animal experiments, there was no need for the approval of the Ethical Committee.

The dogs were required to gently fast (about 8 h) before routine blood sampling to avoid lipemia in collected samples. Peripheral blood samples were taken by cephalic or saphenous venipuncture into tubes with dipotassium ethylenediaminetetraacetic acid (K2-EDTA). Routine biochemistry tests were undertaken on patients dependent on their clinical investigation. Lipemia or hemolysis in serum samples was a rejection criterion. Hematological analysis was performed on all dogs as a part of their qualifying evaluation. A complete blood count was performed (ProCyte DxHaematology Analyser, IDEXX, Westbrook, ME, USA), and blood smears were examined with a CX21 light microscope (Olympus, Tokyo, Japan) after May–Grünwald Giemsa staining. The excess amount of blood samples used for hematology testing was utilized to determine the influence of CLBL-1 EVs and CLB70 EVs on peripheral blood mononuclear cells (PBMCs) after 5 days of co-culture. Blood smear examination and cell culture were performed at the Department of Pathology and Veterinary Diagnostics at the Institute of Veterinary Medicine, Warsaw University of Life Sciences (WULS-SGGW), Warsaw, Poland.

### 4.2. Isolation and Staining CLBL-1 EVs and CLB70 EVs

Two canine cancer cell lines CLBL-1 (B cell lymphoma cell line) and CLB70 (B cell chronic lymphocytic leukemia), were used in this study. CLBL-1 was obtained from Barbara C. Ruetgen, Institute of Immunology, Department of Pathobiology, University of Veterinary Medicine, Vienna, Austria [55], and CLB70 was obtained from Aleksandra Pawlak, Department of Pharmacology and Toxicology, Faculty of Veterinary Medicine Wroclaw University of Environmental and Life Sciences, Wrocław, Poland [18].

CLBL-1 and CLB70 cell lines were maintained in RPMI 1640 with GlutaMAX™ (Gibco, Life Technologies, Bleiswijk, The Netherlands) containing 20% heat-inactivated fetal bovine serum (FBS), penicillin (100 IU/mL), streptomycin (100 μg/mL), nonessential amino acids (1%), MEM vitamins (100 μM), sodium pyruvate (1 mM), and amphotericin B (1 μg/mL) (Gibco™, Life Technologies, Bleiswijk, The Netherlands). The cells were cultured in 25 cm^2^ flasks (Corning Inc., Corning, NY, USA) and passages every second/third day to maintain optimal density (about 70% of cell density). After 7 to 10 days of cell culturing, TEVs were prepared from the supernatant.

To obtain CLBL-1 EVs and CLB70 EVs we used a modified centrifuge-based protocol for isolation of EVs from cell cultures [56,57]. Cells were isolated and removed by pelleting with a centrifuge at 500× *g* for 5 min. The supernatant was carefully transferred to another tube. The next centrifuge step (1200× *g* for 10 min) eliminated the majority of apoptotic bodies in the pellet. Next, the supernatant was centrifuged (22,000× *g* for 40 min) to pelleted TEVs. To minimize protein degradation, centrifuge steps were taken at 4 °C. Subsequently, the pellet was aliquoted in dPBS (sterile and filtered through 0.1 µm membrane filters (Corning, NY, USA)) and frozen at −80 °C until use. TEV protein concentration was measured by a BCA Pierce™ BCA Protein Assay Kit (Thermo Fisher Scientific, Waltham, MA, USA; 23225) according to the manufacturer’s protocol. Absorbance at 562 nm was measured by a BioTek Synergy H1 multiplate reader (BioTek Instruments GmbH, Bad Friedrichshall, Germany).

To evaluate the uptake of CLBL-1 EVs and CLB70 EVs by PBMCs, TEVs were stained with a 5 μM Violet dye from a CellTrace Violet proliferation kit (Life Technologies, Bleiswijk, The Netherlands) for 30 min at RT, protected from light. To eliminate the unincorporated stain, one centrifugation step (22,000× *g* 45 min at 4 °C) was completed [58,59]. Next, TEV pellets were resuspended in the completed cell medium and added to PBMCs. The cells were incubated at 37 °C with 5% CO_2_ and harvested after 30, 60, and 120 min. Finally, the cells were stained with mAbs as described above [12].

### 4.3. PBMC Isolation and Culture

The cells were isolated in sterile conditions from fresh K2-EDTA whole blood by density gradient centrifugation. Histopaque 1077 (Sigma-Aldrich, Germany) was used for the separation of peripheral blood mononuclear cells (PBMCs) according to the manufacturer’s instructions: about 3 mL of blood, gently mixed with 3 mL of buffer (dPBS) (Gibco, Life Technologies, Bleiswijk, The Netherlands) at RT, was layered on 3 mL of Histopaque 1077 in a sterile, V-bottom tube and then centrifuged (400× *g*) for 25 min at RT, without a break (MPV-260R; MPW med. instruments, Warsaw, Poland). The carefully collected fraction of PBMC was washed with RPMI 1640 with GlutaMAX^TM^ (Gibco, Life Technologies, Bleiswijk, The Netherlands), followed by centrifugation for 5 min at RT (400× *g*) and resuspended in 2 mL of completed medium: RPMI 1640 with GlutaMAX™ (Gibco, Life Technologies, Bleiswijk, The Netherlands) containing 10% heat-inactivated fetal bovine serum (FBS), penicillin (100 IU/mL), streptomycin (100 μg/mL), nonessential amino acids (1%), MEM vitamins (100 μM), sodium pyruvate (1 mM), and amphotericin B (1 μg/mL) (Gibco™, Life Technologies, Bleiswijk, The Netherlands). The following day, sample cellularity and cell viability were assessed in an EVE^TM^ cell counter (NanoEntek, Seoul, Korea). A total of 2 × 10^6^ freshly isolated PBMC was cultured in the absence or presence of CLBL-1 EVs or CLB70 EVs in two concentrations (5 or 20 μg/mL) with concanavalin A (ConA) (Sigma-Aldrich, St. Louis, MO, USA; 5 μg/mL). After 24 h, the cells were washed and recombinant canine IL-2 (R and D Systems, Abingdon, UK; 1 ng/mL) was added, and then the cells were incubated for another 4 days. All the cells were incubated at 37 °C with 5% CO_2_. The cultured cells were then used to evaluate the influence of CLBL-1 EVs or CLB70 EVs on the proliferation of lymphocytes, in addition to antigen expression and intracellular cytokine production. On the fifth day, the cells were restimulated with phorbol 12-myristate 13-acetate (PMA) and ionomycin (eBioscience^TM^ Cell Stimulation Cocktail, Invitrogen^TM^, USA; 5 µg/mL). In addition, samples for cytokine production were incubated with BD GolgiStop Protein Transport Inhibitor with Monensin (BD, USA) in sterile conditions at 37 °C with 5% CO_2_.

### 4.4. Cell Staining

Samples with suspended cells planned for the determination of cell proliferative capacity were supravitally stained with a CellTrace™ Violet Cell Proliferation Kit (Life Technologies, Bleiswijk, The Netherlands), before supplementation of CLBL-1 EVs or CLB70 EVs and stimulation and culture of ConA, according to the manufacturer’s instructions.

The surface markers used for phenotyping lymphocytes were canine-specific monoclonal antibodies (mAbs) and had documented cross-reactivity, as included in Table 1.

The appropriate amount of each mAb was determined experimentally to obtain optimal labeling results. The controls included unlabeled cells, and when necessary, FMO (fluorescence minus one) controls were used. In order to block nonspecific mAbs binding, an additional step with 10% BSA (15 min at 4 °C) before staining with antibodies was used. The cells were incubated and protected from light with mAbs for 20 min at RT in eBioscience™ Flow Cytometry Staining Buffer (Life Technologies, Bleiswijk, The Netherlands). Next, cells were washed twice with 2% BSA and resuspended in a 200 μL flow cytometry staining buffer, and immediately introduced into the cytometer.

For tubes with mAbs-CD45RA, a two-step staining procedure was used. After the washing step with 2% BSA, the cells were incubated with IgG1 conjugated with PE (20 min at 4 °C). Lastly, the cells were washed with 2% BSA and resuspended with a 200 μL flow cytometry staining buffer, and immediately introduced into the cytometer.

For intracellular staining of IL-17A and IFN-γ, after surface mAbs staining, cells were incubated with the permeabilization solution (20 min at RT, protected from light) (BD Cytofix/Cytoperm™, BD, USA). Next, after the washing step, the cells were incubated with IL-17 and IFN-γ mAb (30 min at 4 °C in the dark), washed, and then resuspended with a 200 μL flow cytometry staining buffer and immediately introduced into the cytometer.

### 4.5. Flow Cytometry Analysis

The gating strategy was previously described (Zmigrodzka et al., 2022) and is shown in Supplementary Figure S3. Doublets were eliminated from the analysis by setting the region on single cells on the FSC-area (FSC-A) vs. FSC-high (FSC-H) dot plot. Next, the lymphocytes were gated based on FSC and SSC dot plots. In the T-lymphocyte, region analyses of CD4^+^ and CD8^+^ cells with co-expression of CD45RA and CD62L were performed for naïve, CM, EM, and TEMRA Th and TC lymphocytes. The next samples included CD4^+^ and CD8^+^ cells with co-expression of intracellular IL-17 and IFN-γ. Cell proliferation intensity was calculated from singlets for lymphocytes T (CD3^+^) and B (CD3^−^CD21^+^). A flow cytometric analysis was performed using a FACSCanto II flow cytometer and Diva software (Becton Dickinson, Franklin Lakes, NJ, USA); 20,000 cells of each sample were acquired. Prior to multicolor staining, the compensation was set using single-positive cells for each color.

### 4.6. Statistical Analysis

Statistical analysis was performed in Prism software, version 5.0 (GraphPad Software, San Diego, CA, USA). One-way ANOVA and Tukey’s HSD post hoc test were applied to determine the statistical significance of control cells (not TEV-treated) and TEV-treated cells between different concentrations of the TEVs. A * *p*-value < 0.05 was regarded as significant, whereas a ** *p*-value < 0.01 and *** *p*-value < 0.001 were highly significant.

## 5. Conclusions

Canine PBMCs show different phenotype and secretion properties as a result of TEV stimulation. The higher percentages of CD3^+^ and CD4^+^ TEMRA cells, and decreased B, IL-17, and CD8^+^ lymphocytes after co-culturing with two concentrations of CLBL-1 EVs and CLB70 EVs, may indicate their immunomodulatory potential. Future research should focus on assessing changes in T and B regulatory cells and the quantitative contribution at different time points of TEV stimulation, and determining other differences in their activity. To confirm the current findings, further research is necessary, taking into account the role of TEVs in cancer development.

## Figures and Tables

**Table 1:** Monoclonal antibodies used in the study for labeling peripheral blood mononuclear cells (PBMCs) for flow cytometry. Abbreviations: FITC—fluorescein isothiocyanate, PE—phycoerythrin, AF647—Alexa Fluor 647, PB—Pacific Blue.

Antibody	Clone	Isotype	Host Species	Fluorochrome	Dilution
CD4:CD8	YKIX302.9/YCATE55.9	IgG2a	rat	FITC:PE	1:5
CD4	YKIX302.9	IgG2a	rat	AF647	1:5
CD8	YCATE55.9	IgG1	rat	FITC	1:5
CD3:CD8	CA17.2A12/YCATE55.9	IgG1	mouse	FITC:PE	1:5
CD21	CA2.1D6	IgG1	mouse	AF647	1:5
CD45RA	CA4.1D3	IgG1	mouse	-	1:5
CD62L	FMC46	IgG2b	mouse	PB	1:5
IL-17A	eBio17B7	IgG2a,κ	rat	PE	1:20
IFNγ	CC302	IgG1	mouse	AF647	1:5
IgG1	M1-14D12	IgG1	rat	PE	1:5

## Data Availability

The data presented in this study are available on request from the corresponding author.

## Supplementary Materials

The supporting information can be downloaded at: https://www.mdpi.com/article/10.3390/ijms23179831/s1.
