# Supplementary figures and images for: Canine B Cell Lymphoma- and Leukemia-Derived Extracellular Vesicles Moderate Differentiation and Cytokine Production of T and B Cells In Vitro
Source: Int J Mol Sci. 2022 Aug 29;23(17):9831. doi: 10.3390/ijms23179831 (PMC9456052; doi:10.3390/ijms23179831)

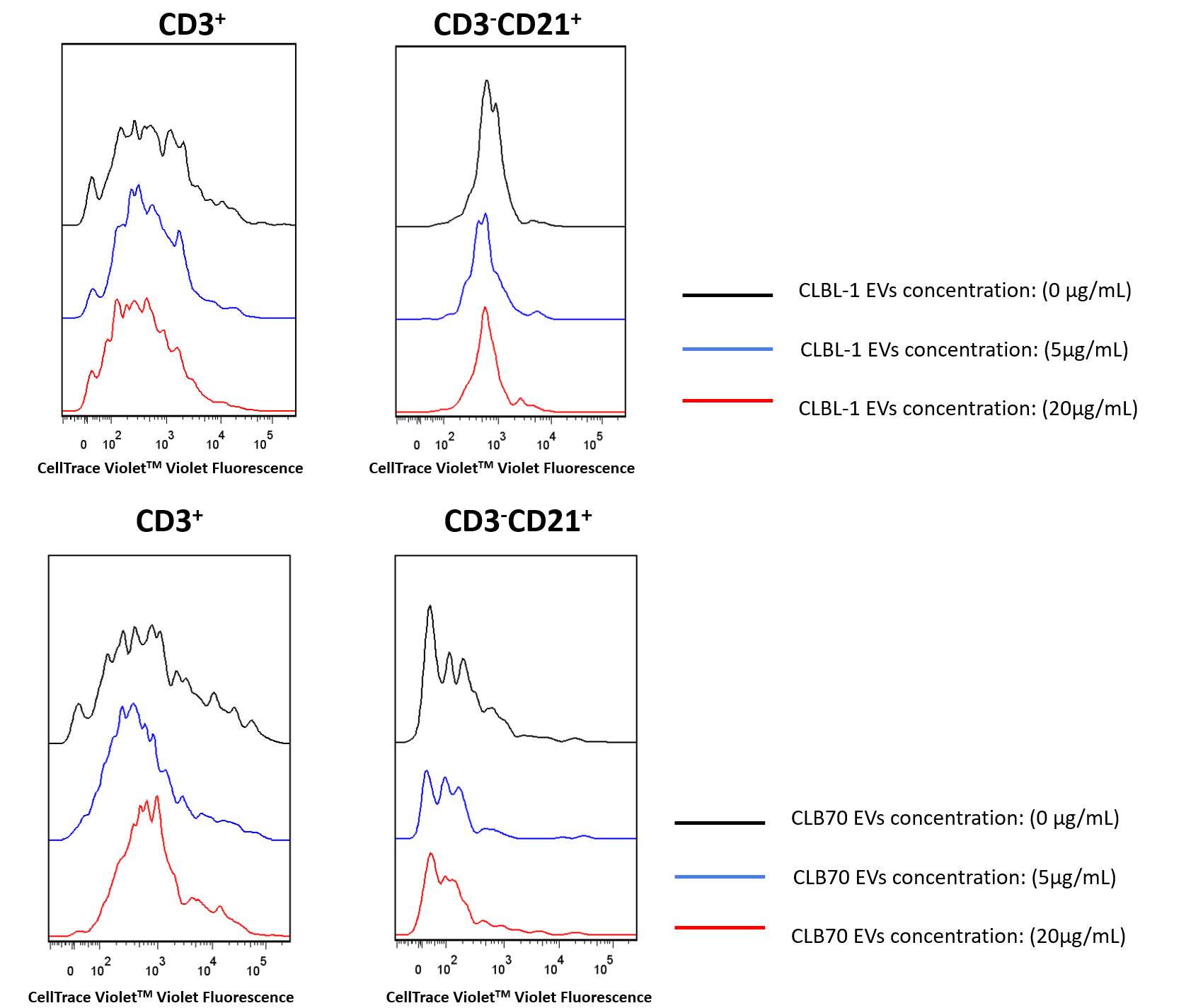

Supplement: Supplementary file 1 [file ijms-23-09831-s001.zip › Supplementary FigureS1.jpg]

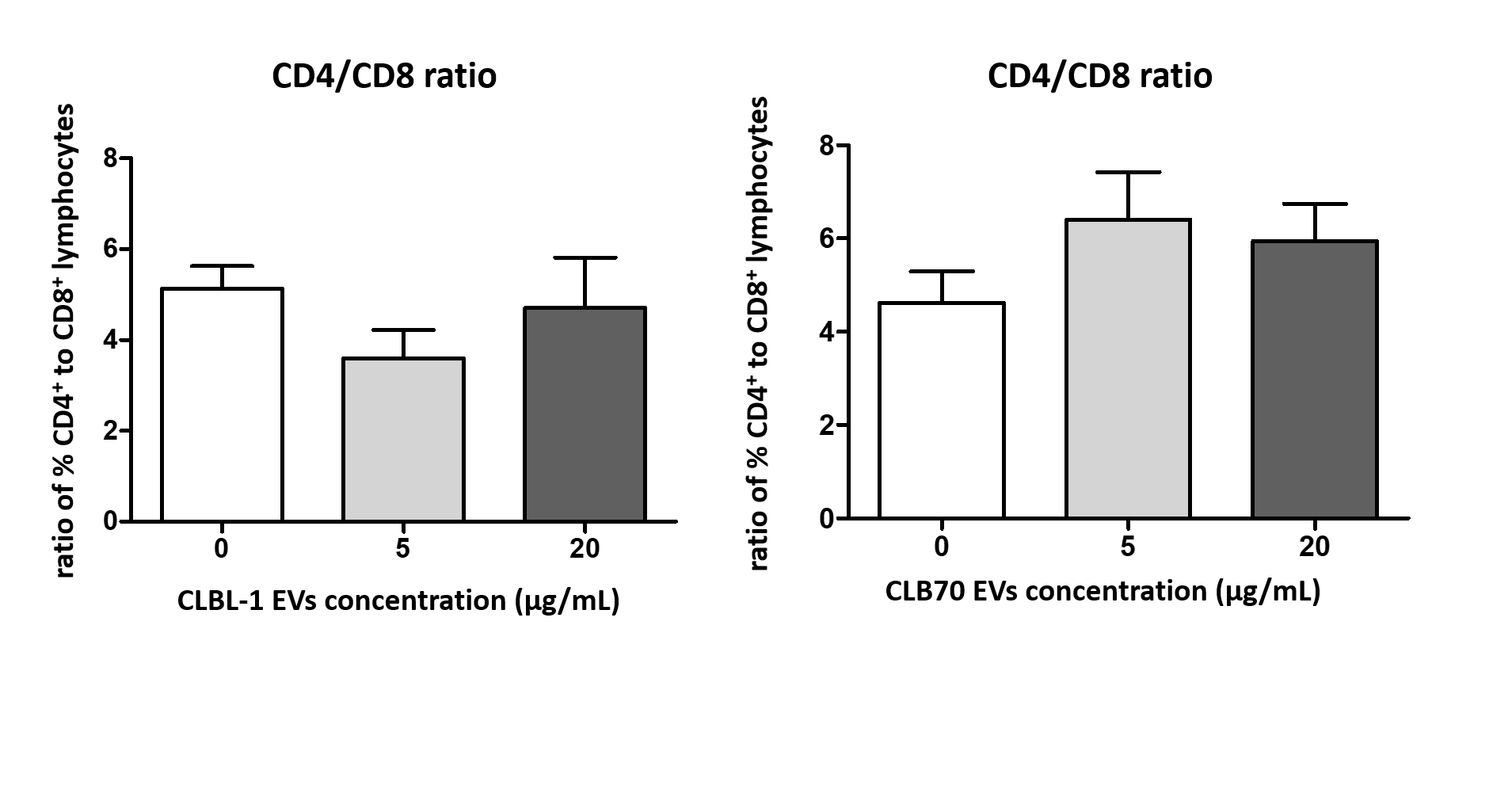

Supplement: Supplementary file 1 [file ijms-23-09831-s001.zip › Supplementary FigureS2.jpg]

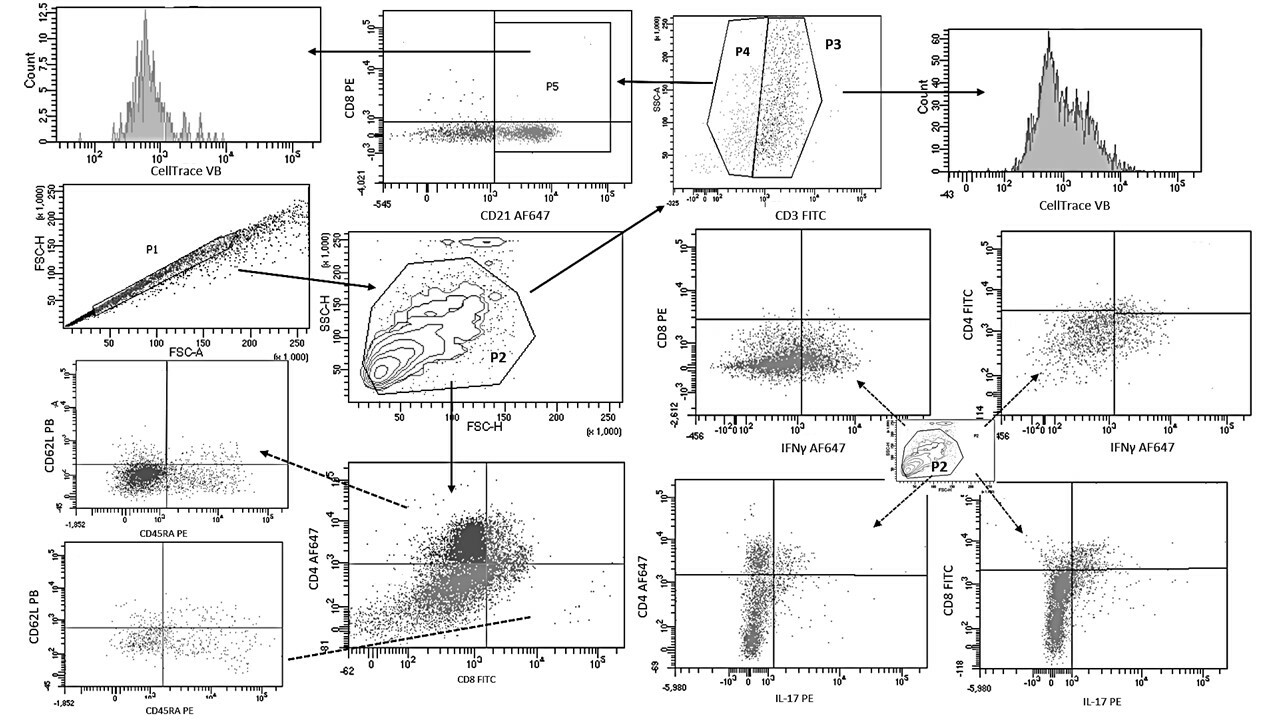

Supplement: Supplementary file 1 [file ijms-23-09831-s001.zip › Supplementary FigureS3.jpg]
